# Embryo selection: the role of time-lapse monitoring

**DOI:** 10.1186/1477-7827-12-124

**Published:** 2014-12-15

**Authors:** Peter Kovacs

**Affiliations:** Kaali Institute IVF Center, Istenhegyi u. 54/a, 1125 Budapest, Hungary

**Keywords:** Embryo selection, Time-lapse monitoring, Embryo culture, Implantation, Single embryo transfer

## Abstract

In vitro fertilization has been available for over 3 decades. Its use is becoming more widespread worldwide, and in the developed world, up to 5% of children have been born following IVF. It is estimated that over 5 million children have been conceived in vitro. In addition to giving hope to infertile couples to have their own family, in vitro fertilization has also introduced risks as well. The risk of multiple gestation and the associated maternal and neonatal morbidity/mortality has increased significantly over the past few decades. While stricter transfer policies have eliminated the majority of the high-order multiples, these changes have not yet had much of an impact on the incidence of twins. A twin pregnancy can be avoided by the transfer of a single embryo only. However, the traditionally used method of morphologic embryo selection is not predictive enough to allow routine single embryo transfer; therefore, new screening tools are needed. Time-lapse embryo monitoring allows continuous, non-invasive embryo observation without the need to remove the embryo from optimal culturing conditions. The extra information on the cleavage pattern, morphologic changes and embryo development dynamics could help us identify embryos with a higher implantation potential. These technologic improvements enable us to objectively select the embryo(s) for transfer based on certain algorithms. In the past 5-6 years, numerous studies have been published that confirmed the safety of time-lapse technology. In addition, various markers have already been identified that are associated with the minimal likelihood of implantation and others that are predictive of blastocyst development, implantation potential, genetic health and pregnancy. Various groups have proposed different algorithms for embryo selection based on mostly retrospective data analysis. However, large prospective trials are needed to study the full benefit of these (and potentially new) algorithms before their introduction into daily practice can be recommended.

## Background

In vitro fertilization (IVF) is one of the fastest developing fields of medicine. The first birth following successful treatment was reported in 1978 [1]. According to a 2014 report, in 2005, over 1 million treatment cycles had been performed worldwide, and in just that year, more than 250,000 children born were conceived in vitro [2]. As of now, it has been estimated that over 5 million children have been born as a result of IVF.

Despite the technical developments of the last decades, there are still problems associated with the treatments that need to be solved. While many aspects of in vitro fertilization have improved significantly, the still relatively low implantation rate often results in the transfer of several embryos and leads to multiple gestations.

In 2009, over 400,000 fresh IVF, intracytoplasmic sperm injection (ICSI) cycles were performed in Europe. One out of every fifth treatment resulted in a live birth. To achieve this birth rate, more than 1 embryo was transferred in over 75% of the cycles. Twenty percent of the deliveries involved multifetal pregnancies [3]. In 2012, close to 100,000 fresh IVF/ICSI cycles were performed in the US. The average number of embryos transferred (depending on the age of the patient) was between 1.9 and 2.9. The implantation rate was 37.5% in the youngest (<35) age group. In this same age group, a 40.7% live birth rate was achieved but almost 30% of the deliveries were twin deliveries [4].

A multiple gestation, even a twin pregnancy, is known to carry extra maternal and neonatal risks [5] Table 1.

**Table 1:** **Maternal and perinatal risks associated with preterm deliveries**

Multiple pregnancy	
Maternal risks	Neonatal risks
Gestational hypertension, preeclampsia, eclampsia, gestational diabetes, thromboembolism, postoperative hemorrhage, operative delivery, etc.	Preterm delivery, prematurity, delayed motor-, neuro development respiratory distress, intraventricular hemorrhage, infection, retinopathy, gastrointestinal problems, anomalies
Increased morbidity, mortality	Increased morbidity, mortality, long-term health consequences requiring long-term care
Significant increase in health care expenses	

To address the problem of multiple gestations following IVF, more restrictive transfer policies have been recommended and various methods have been studied that could help embryo selection [6, 7].

Single embryo transfer could essentially eliminate multiple pregnancies. It has been shown that the transfer of a single embryo reduces the multiple pregnancy rate by over 90%, but the pregnancy rate is reduced by close to 50% as well [8, 9]. However, when a failed single fresh transfer is followed by the transfer of a single frozen-thawed embryo, the outcome is similar to that following a double fresh embryo transfer (single fresh + single frozen ET: 38% vs. double ET: 42%; OR: 0.85; 95% CI: 0.62-1.15) [8].

The ability of various screening technologies (preimplantation genetic screening, metabolomics, proteomics) to identify embryo(s) with the highest implantation potential has been evaluated in recent years. Some of these methods require the use of complicated technology, necessitate elective embryo cryopreservation and are associated with significant treatment expenses. In addition, most randomized trials do not support their use [10–13]. Preimplantation genetic screening using array comparative genomic hybridization has been shown to improve clinical outcome in young, high responder patients [14, 15]. Time-lapse monitoring is another tool that has been evaluated as an aid to identify the embryo(s) with the highest implantation potential.

## Review

### Time-lapse monitoring

Usually the embryologists remove embryos from the incubator once per day to assess cleavage and morphology, but this type of monitoring only gives them a snapshot of a dynamic process. The embryos do not tolerate removal from optimal culturing conditions, which limits the number of observations that can be made. This problem is a significant one for the embryologists, and time-lapse technology may offer a solution. With this technology, the embryos can be monitored without removing them from the incubator. A camera is built into the incubator and takes pictures of the embryos at preset intervals. With the help of the proper software, a video can be made that depicts their development. This type of monitoring allows for the collection of much more information on the timing of the cleavages and the dynamics of the morphologic changes. Payne and colleagues [16] were among the first to describe the early events of human embryonic development, and then, Mio and Meada described the kinetics of the events up until the blastocyst stage [17]. Their work was followed by observations made by several other groups that tried to correlate these kinetic and morphologic markers with embryo development, implantation potential, pregnancy rate and genetic health. This review will summarize the findings of these publications.

### Time-lapse technology

Various time-lapse systems are currently used. Two of the most widely used technologies, the Primo Vision (Vitrolife) [18] and Embryoscope (Fertilitech) [19] systems, both use bright field technology, whereas the EEVA (Early Embryonic Viability Assessment, Auxogyn) system uses dark field technology [20]. All systems incorporate a digital inverted microscope that takes a picture of the embryos at 5-20 minute intervals. The images are processed by custom image acquisition and then displayed on a computer screen. The pictures taken at preset intervals are then connected into short films that can be rewound and fast forwarded for detailed analysis.

The Embryoscope [19] is an incubator with an integrated time-lapse system, where the embryos, cultured individually in microwells, are moved one by one into the field of view of the inbuilt microscope at each of the image acquisitions. In the Embryoscope system, embryos are cultured in special culture dishes (Embryoslide, Fertilitech). This multi-well dish allows the monitoring of up to 12 individually cultured embryos. The Embryoscope can follow 6 of these dishes (max. 72 embryos) simultaneously. It takes pictures every 12-20 minutes and can evaluate the embryos in 7 focal planes. It uses low intensity red LED illumination (635 nm) with <0.5 secundum per image light exposure.

Primo Vision [18] is a compact digital inverted microscope system that is designed to be placed inside of existing small- to large-sized traditional incubators. Control of the system, patient database build-up, embryo development analysis and decision-making are performed outside of the incubator through a controlling unit. Embryos in the Primo Vision system are also cultured in multi-well dishes (Primo Vision embryo culture dish, Vitrolife) that contain 9-16 wells. However, embryos in this system are covered by a single drop of culture medium. This system allows individual embryo observation while maintaining the benefits of group culture. The Primo system can monitor up to 16 embryos from the same patient. The units (maximum of 6) are connected with a controlling unit that is outside the incubator with a USB connection. The system uses low intensity green LED (550 nm) illumination and is also able to evaluate the embryos in up to 11 focal planes. Each controlling unit is able to follow a maximum of 96 embryos at the same time.

Like the other systems, the EEVA system [20] requires a special microscope to be placed in the incubator. This system uses dark field illumination to better outline the cell membranes. Embryos are cultured in the specially designed EEVA dish. Based on the timing of the early cleavage events up until the 4-cell stage, the software selects the embryos that are most likely to develop to the blastocyst stage. Table 2 summarizes the main characteristics of the 3 different systems.

**Table 2:** **Comparison of the technical parameters of three commercially available time-lapse systems**

	Embryoscope	Primo vision	EEVA
Illumination	Bright field, low intensity red LED	Bright field, low intensity green LED	Dark field
Microscope/incubator	Incubator with integrated time-lapse system	Microscope that can be placed in standard incubators	Microscope that can be placed in standard incubators
Culture dish	Embryoslide	9-16 well Primo vision embryo culture dish	EEVA dish
Embryo culture	Single culture	Group culture	Group culture
Planes of view	7 focal planes	11 focal planes	Single plain
Max.# of embryos monitored	72	96	Depends on the dish
Other	Comes with software	Comes with software	Automated, software scores blastocyst formation potential

The three systems differ in the way that they observe embryos. In the Embryoscope, the tray holding the culture dishes is under constant movement to bring each embryo individually into the field of view. When the tray is fully loaded (72 embryos), it takes 20 minutes until the next image of a given embryo is taken. This interval does not allow the embryologist to detect rapid changes accurately (e.g., S1 which should last <30-35 min). The constant movement, electromagnetic effects, heat and volatile organic compounds released from the lubricants related to this technology carry the potential to exert adverse effects though no such negative effect has been directly confirmed yet. However, this technology enables the system to maximize resolution. Each Primo Vision microscope is able to monitor up to 16 embryos at the same time without moving them. With this method, the embryos are cultured in a completely undisturbed environment. This system requires significantly less frequent image acquisitions (because all 16 embryos are observed at the same time); hence, the exposure to light, electricity and electromagnetic effects is even lower than that possible with the Embryoscope. This technology, however, does not provide us with the same image resolution. It needs to be emphasized that the light exposure compared to the current standard light microscope evaluation is significantly reduced with both systems.

The EEVA system uses dark field illumination, which allows more accurate observations of the blastomere membranes; therefore, divisions can be monitored accurately but the method gives far less information regarding intracellular morphology and has limited ability to follow embryos beyond day 2 with increasing number of cells. The automated system could confuse large fragments with blastomeres, which could therefore affect its selection precision. The dark filed technology, however, exposes the embryos to significantly higher light load compared to the other two systems. The EEVA system comes with software that predicts which embryo is most likely to turn into a blastocyst based on observations of early markers by day 2 of development. The use of the EEVA system has been shown to decrease inter-observer variability and increase the embryologist’s ability to correctly identify the best embryos [21].

### How does time lapse monitoring help embryo selection?

The current standard in most laboratories is to use morphology for embryo selection. Based on morphologic characteristics, embryos can be scored at various stages (pronuclear, cleavage, blastocyst) [22, 23]. However, it is recognized that this approach is far from being perfect as an average of only approximately 20-40% of the embryos identified this way will implant [3, 4].

It has been previously reported, based on the standard morphologic assessment, that earlier cleaving embryos have a better chance to develop into blastocysts and implant [24]. It was also noted that embryos that reach the blastocyst stage are less likely to be aneuploid, and implantation rates are higher when blastocysts are transferred [25]. Therefore, many centers use extended culture to the blastocyst stage and perform the transfers on day 5 after retrieval. This practice, however, adds to the work of the embryologist, increases the costs associated with embryology procedures and may be associated with adverse effects due to epigenetic changes, though the data are sparse to support such an effect [26, 27]. On the other hand, blastocyst stage transfer has been shown to result in about a 40% increase in pregnancy rates when compared to cleavage stage transfer [28]. This improvement may be due to better embryo selection or improved embryo-endometrium synchrony. Therefore, despite the slightly higher cost of the cycle with blastocyst transfer, it may save money in the long-run by reducing the number of cycles that have to be performed.

Time-lapse technology is expected to improve the embryologist’s ability to select the embryo with the highest implantation potential (even by day 3 or at the blastocyst stage), and this improvement should be translated into an improved clinical outcome. Automated systems that identify the embryo(s) to be transferred with the help of a software program also ease the embryologist’s work.

**Figure 1:**
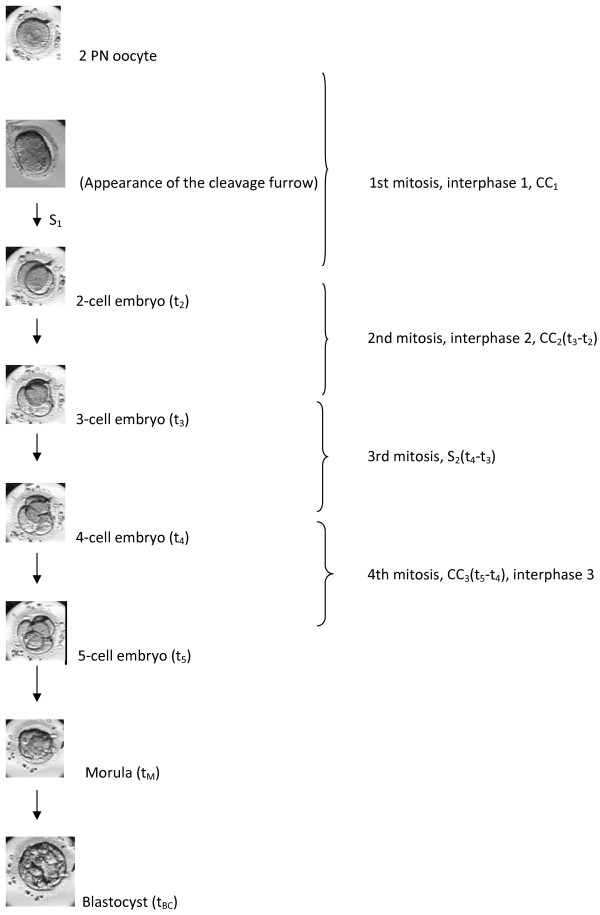
**Embryo development from 2PN to blastocyst stage and the various terminology used in the different papers for certain developmental events.**

Before the results of various studies are discussed, the terminology used by the different papers must be reviewed. Unfortunately various papers use different terminology that can be confusing, as discussed by Kirkegaard and et al. [29]. Mitotic events in the fertilized egg will increase the cell number, and each mitosis will result in the formation of two cells from the precursor cell. Therefore, the first mitosis will result in a two-cell embryo, the second mitosis in a three-cell embryo and so on. However, the cell number doubles as the embryo passes through the cell cycles roughly every 24 hours. The second cell cycle involves two mitoses: one that will turn a two-cell embryo into a three-cell embryo and a second one that will turn a three-cell embryo into a four-cell embryo. On day 4, the embryo should reach the morula stage and by day 5, the blastocyst stage. In these stages, the cell number can no longer be followed, but morphologic changes can be used to identify them. In papers evaluating time-lapse technology, the use “cell cycle” and “cleavage cycle” terminology is often confusing. A cell cycle will result in two cells while a cleavage cycle will result in doubling of the cells within the embryos. Figure 1 summarizes the events up until the blastocyst stage and explains the various nomenclature, including a description of certain early events used in the different publications (Figure 1).

### Observations made using time-lapse technology

In a study published in 1997, Payne and colleagues [16] described their observations based on time-lapse imaging of 50 fertilized oocytes. Oocytes were randomly selected for time-lapse imaging, which was initiated 30 minutes following intracytoplasmic sperm injection (ICSI) fertilization. Three quarters of the oocytes time-lapse observed fertilized successfully; this rate did not differ from the sibling oocytes followed under standard conditions. The proportion of embryos that were considered good quality embryos on day 3 was also similar between the two methods. Time-lapse observation noted marked variation in the timings of polar body extrusion, pronuclear formation and abuttal among the oocytes, and significant differences were noted between oocytes that formed good versus poor quality embryos by day 3. In addition, time-lapse technology was used to observe several cytoplasmic events, such as cytoplasmic waves, during the observations made in the first 24 hours following fertilization.

Lemmen et al. [30] evaluated 102 2PN oocytes using time-lapse monitoring. Fertilized eggs were randomly assigned to time-lapse versus standard observation. By day 2, 58% of the time-lapse observed embryos had developed to at least the 4-cell stage. A total of 82% of the fertilized oocytes developed into embryos that were suitable for transfer or cryopreservation. This rate was similar to that obtained in standard incubators. Implanted embryos had a faster and more synchronous appearance of the nuclei after the first cleavage. Those embryos that reached the 4-cell stage by day 2 had a faster disappearance of the pronuclei. Embryos with more cells (≥ 4 cells) on day 2 had a faster first cleavage when compared to embryos with fewer than 4 cells on day 2. However, specific time intervals for certain early events to occur were not provided.

Azzarello and colleagues studied whether pronuclear stage changes are predictive of live birth. A total of 159 embryos that were transferred on day 2 were analyzed in a prospective manner, and 46/159 embryos resulted in live birth. In contrast to the observations made by Lemmen et al. [30], this group found that pronuclear breakdown occurred significantly later in zygotes that resulted in live birth when compared to zygotes that failed to result in live birth. None of the embryos in which the pronuclear breakdown occurred before 20 hours and 45 minutes successfully implanted. Several pronuclear scoring systems were evaluated, but none of them were found to be predictive of live birth [31].

Wong et al. [32] studied the developmental kinetics of frozen/thawed fertilized oocytes (n = 242) that had been donated to research. Various kinetic parameters (for 100 of the 242 embryos) as well as gene expression profiles (for 142 of 242 embryos) of embryos that reached the blastocyst stage by day 5 (normal) versus those that did not reach the blastocyst stage (abnormal) were compared. A total of 33-53% of the 100 embryos cultured to day 5 or 6 reached the blastocyst stage (a range is given as there were several experimental sets). Three parameters were significantly predictive of blastocyst development: first cytokinesis (mean 14.3 ± 6 min), time between 1st and 2nd mitosis (mean: 11.1 ± 2.2 hrs), time between 2nd and 3rd mitosis (mean: 1 ± 1.6 hrs). An automated model to predict blastocyst formation was developed using these parameters and was tested on a subset of 14 embryos. All eight embryos that reached the blastocyst stage were correctly identified by the model. Abnormal embryos fell out of the optimal cleavage time ranges or had multiple cleavage furrows and displayed significant fragmentation. As none of the embryos were transferred, these identified early kinetic markers could not be used to determine improved embryo implantation or pregnancy.

Meseguer et al. [33] studied the kinetics of embryo development and implantation potential based on the retrospective analysis of 247 time-lapse embryo observations in which the implantation for all transferred embryos was known (0 or 100% implantation). Direct cleavage from 1 → 3 cells (very short time as a 2-cell embryo; [< 5 hrs]), uneven blastomeres at the 2-cell stage (>25% size difference) and multinucleation at the 4-cell stage have been associated with a minimal chance that these embryos will implant and were suggested as exclusion criteria by the authors (exclusion from transfer). Furthermore, they divided each kinetic parameter into time range quartiles. Embryos with cleavage times (t2,3,4,5) that fell into the 2 middle quartiles (considered optimal range) were more likely to implant compared to embryos with cleavage times that fell outside this range. Out of these parameters, cleavage to the 5-cell stage (t5) within the optimal range was the most predictive of implantation. When cell cycle parameters were analyzed, embryos with a CC2 (2 → 3 cell division) and S2 (3 → 4 cell division) that fell into the first two quartiles were most likely to implant. Therefore, these time ranges were determined as optimal. Logistic regression analysis identified t5 as the most predictive (followed by S2 and CC2) of implantation. These criteria were then used to develop a hierarchical model for embryo selection. Embryos with direct cleavage, multinucleation or uneven blastomeres at the 2-cell stage were excluded (category E). For those embryos not excluded, t5 was evaluated (optimal range 48.8-56.6 hrs), followed by S2 (optimal range: ≤ 0.76 hrs) and then CC2 (optimal range: ≤ 11.9 hrs). Eight categories were created based on whether the embryo fell into the optimal range or not for the three parameters. The embryos with the highest chance to implant fell into the optimal range for all three parameters.

The significance of the exclusion criteria (direct cleavage from 1 → 3 cells) established by Meseguer et al. [33] were further evaluated by Rubio and colleagues [34]. Embryos were considered to cleave directly when the 2-cell stage was <5 hrs (CC2). The analysis was based on 1,659 transferred embryos. The overall implantation rate was 29.2%. A total of 109 of the transferred 1,659 embryos were identified as direct cleaving embryos. Only 1 (1.2%) of the direct cleaving embryos was known to implant. The implantation rate of the non-direct cleaving embryos (CC2 > 5 hrs) with known implantation was 20.2%.

In a retrospective analysis based of 834 embryos, Cruz and colleagues [35] assessed whether time-lapse kinetic parameters can be used to predict blastocyst formation. For time-lapse categorization, the t5 and S2 time ranges identified by Meseguer et al. were used, but further kinetic markers were compared between embryos that reached the blastocyst stage versus those that did not. Four categories were established based on t5 and S2: A: t5 and S2 in the optimal range; B: t5 in the range, while S2 out of the optimal range; C: t5 out of the optimal range, while S2 in the optimal range; and D: t5 and S2 both out of optimal range. Embryos with uneven blastomeres at the 2-cell stage and those directly cleaving from 1 → 3 cells were less likely to turn into blastocysts or to turn into a blastocyst with good morphology, thereby confirming previous observations. Cleavage times (t2, t3, t4), CC2, S2 and time to the morula stage were significantly shorter for those embryos that had reached the blastocyst stage. T5 however was similar between those embryos that turned into blastocysts versus those that did not. When a distinction was made based on blastocyst morphology, stages t4, t5, s2 and the time to morula stage were shorter among blastocysts with good morphology when compared to poor quality blastocysts. The implantation status was known for 120 embryos (0 or 100%). The implantation rates were not significantly different among the 4 categories identified based on t5 and S2 (A: 51.6%, B: 64.2%, C: 34.3%, D: 30%). This study has shown that kinetic markers are predictive of blastocyst formation and the development of blastocysts with good morphology, but the hierarchical categorization based on t5 and S2 previously proposed by Meseguer et al. was not able to predict implantation. The authors offer the relatively small sample size as a possible explanation for this result.

In a separate retrospective analysis based on 7,305 cycles, the Meseguer lead group [36] compared the pregnancy outcome between embryos cultured in standard incubators (N = 5915) or time-lapse systems (N = 1390). Embryos cultured in the time-lapse system were scored according to their previously established hierarchical model based on t5, S2 and CC2. Data were obtained from 10 participating clinics. A higher pregnancy rate (+21.2%) was achieved with the transfer of embryos cultured in the time-lapse system. Logistic regression analysis was used to correct for confounding variables. Using the fully corrected model, an increase of 20.1% based on oocyte retrieval and 15.7% based on embryo transfer was achieved with embryos cultured in the time-lapse system. The authors attributed this increase in success to the undisturbed culture conditions and the ability to select embryos for transfer based on strict morphokinetic criteria.

The retrospective analysis by Chamayou et al. was based on time-lapse observations made during the assessment of 72 implanted, 106 non-implanted and 66 arrested embryos. Based on the analysis of their time-lapse recordings, the following kinetic parameters were associated with development into viable day 5 embryos: t1 (18.4-30.9 hrs), t2 (21.4-34.8), t4 (33.1-57.2), t8 (46.1-98.5 hrs), and s3 [t8-5] (0.7-30.8 hrs). None of these parameters, however, were predictive of successful implantation. The only parameter that was significantly associated with implantation and clinical pregnancy was CC3 (9.7-21.0 hrs) [37].

Kinetic parameters up until the 8-cell stage were compared between embryos that had reached and embryos that had not reached the blastocyst stage in a retrospective analysis by Dal Canto and colleagues. Furthermore, kinetic markers between expanded and non-expanded blastocysts and implanted and non-implanted blastocysts were compared. Embryos that had turned into blastocysts required a shorter time to reach the 8-cell stage (61.0 +/- 9.4 hrs vs. 65.2 +/- 13 hrs), but the difference in timing occurred only after the 6-cell stage. Expanded blastocysts reached all cellular stages faster and progressed to the 8-cell stage on average 4 hours sooner than the non-expanded blastocysts. Finally, implanted blastocysts required a shorter time to reach the 8-cell stage when compared to non-implanted blastocysts (54.9 +/- 5.2 hrs vs. 58.0 +/- 7.2 hrs) [38].

Conaghan and coauthors [21] tested an automated time-lapse analysis system to determine whether it can predict blastocyst formation. Their study was a prospective study in which day 3 morphology alone or day 3 morphology supplemented by early embryo viability assessment [EEVA] to predict blastocyst development were compared. The study involved a development phase in which the predictive ability of early events (P1: first cytokinesis; P2: time between 1st and 2nd cytokinesis [CC2]; P3: time between 2nd and 3rd cytokinesis [S2]) were tested. Data from the test phase showed that when P2 (CC2) fell in the 9.33-11.45 hrs range and P3 (S2) fell in the 0-1.73 hrs range, the probability of developing into a usable blastocyst was higher (positive predictive value: 54.1%; negative predictive value: 86.6%). In the test phase it was determined that EEVA assessment significantly improved the predictive value of usable blastocyst identification compared to morphology alone (54.7% vs. 34.5%). Only the predictive ability for blastocyst formation was reported in this study, but implantation and pregnancy rates were not.

Kirkegaard et al. studied 92 patients with a good prognosis in a prospective cohort study in which they tested the predictive ability of early time-lapse kinetic parameters (during the first 48 hours of development). The predictive ability of high-quality blastocyst development was analyzed based on 571 embryos, whereas pregnancy outcome was evaluated based on 84 single embryo transfers. The duration of the first cytokinesis, duration of the 3-cell stage and the lack of direct cleavage to 3 cells were predictive of high-quality blastocyst development. The kinetic parameters, however, did not significantly differ between implanted and non-implanted embryos [39].

There have been two randomized controlled trials (RCT) published so far that evaluated embryo selection for transfer based on time-lapse parameters. The results of the first trial were published by Kahraman and colleagues [40]. This group randomly assigned the embryos of 64 good prognosis patients to culturing in a time-lapse system or under standard incubation conditions. A single blastocyst was transferred in all cases. The embryo cultured in the conventional incubator was selected for transfer based on day 5 morphology. Embryos cultured in the time-lapse system were selected based on blastocyst morphology and the hierarchical model suggested by Meseguer et al. (t5, S2, CC2). The blastocyst development rate was similar for both incubation methods. Pregnancy (60.6% vs. 61.2% ongoing pregnancy rates) and miscarriage rates were comparable as well. Embryo selection based on time-lapse criteria in addition to morphologic criteria did not further increase the chance of implantation. While kinetic parameters (t 2,3,4,5; time to morula stage; time to blastocyst stage; CC2, S2) did not differ between embryos that implanted (n = 24) and those that failed to implant (n = 9), blastocysts with good morphology differed significantly from poor quality blastocysts regarding these parameters.

The second RCT was published recently by Rubio and colleagues [41]. In this study, 930 good prognosis patients or patients undergoing egg donation were randomly assigned to standard incubation and selection based on morphology alone versus incubation in a time-lapse system and embryo selection based on morphokinteic criteria (Meseguer hierarchical model [33]). The ongoing pregnancy rate based on all started cycles was higher in the time-lapse monitored group (51.4% vs 41.7%; p = 0.005). When the ongoing pregnancy rate per transfer was evaluated, the time-lapse system still offered benefits over the standard incubation method (54.5% vs 45.3%; p = 0.01). This is the only published study so far that was adequately powered to detect a difference in ongoing pregnancy rates between standard incubation methods and embryo culture in a time-lapse system with embryo selection based on morphokinetic parameters. A logistic regression analysis controlling for confounding variables found a 36% increase in ongoing pregnancy rates with the time-lapse system (OR: 1.36; 95% CI: 1.1-1.68) [41].

### Time-lapse parameters and aneuploid

Several groups assessed the association between time-lapse markers and embryo aneuploidy. Seventy-five fertilized eggs donated to research were analyzed by Chavez et al. [42]. Fifty-three of the fertilized eggs started to cleave, and their development was observed using time-lapse technology for 48 hours. At that stage, all blastomeres of the embryos (185 in total) were tested for chromosome content using array comparative genomic hybridization (aCGH). A total of 45 embryos provided conclusive aCGH data; 17.8% of them were euploid, 75.5% aneuploid and 6.5% triploid. Euploid embryos followed a tight pattern regarding early kinetic markers (S1: 14.4+/-4.2 min, CC2: 11.8+/-0.71 hrs, S2: 0.96+/-0.84 hrs). The development of aneuploid embryos was less predictable, the standard deviation around the mean values was much greater and many of them had kinetic parameters outside the optimal range. Chromosomally abnormal embryos were more likely to display fragmentation.

Basile and colleagues [43] also studied the morphokinetic characteristics of chromosomally normal and abnormal embryos. Data were available for 77 embryo transfer cycles in which day 3 embryo biopsy and aCGH analysis were performed. A total of 28.3% of the tested embryos were euploid. Euploid embryos were more likely to fall into optimal ranges for t5, CC3 and t5-2. Embryos with t5 in the optimal range were 2.8 times more likely, whereas embryos with CC3 in the optimal range were twice as likely to be euploid. A total of 36% of those embryos with both t5-2 and CC3 in the optimal range were euploid, whereas only 9.8% were euploid when both values were outside the optimal range.

Campbell and colleagues published two papers [44, 45] about their findings regarding embryo morphokinetic parameters and genetic health. In their first paper, they describe their observations made based on 98 blastocysts from 25 couples. All embryos were followed in time-lapse systems up until day 5 when trophectoderm biopsy was performed and either aCGH or single nucleotide polymorphism microarray was applied for genetic screening. Euploid embryos required a significantly shorter time to the initiation of compaction, time to the start of blastulation (tSB) and to full blastulation (tB). Early cleavage and cell cycle parameters showed no correlation with euploidy. A model was developed based on these observations. According to the model, a low risk for aneuploidy can be expected when tSB <96.2 hrs and tB <122.9 hrs; medium risk can be expected when tB <122.9 hrs and tSB >96.2 hrs and a high risk can be expected when tB >122.9 hrs [44].

In their second paper, they tested this embryo aneuploidy model. The times to the start of blastulation and full blastulation were retrospectively applied in cycles with known implantation. Genetic testing was not performed in these cycles. Overall, 42% of the blastocysts implanted. Those embryos that were identified to be at high risk for aneuploidy (tB > 122.9 hrs) did not implant. In the low risk group (tB < 122.9 hrs and tSB < 96.2 hrs), the live birth rate was 61.1%, which was 56% higher than the live birth rate in the entire group [44]. The observations made by Campbell and colleagues were criticized by Ottolini et al. in their commentary in which they note that the conclusions were drawn based on small number of cases without taking age as a confounding factor into account [46].

### Safety

It is important to establish the safety of any new technology. The periodic light exposure, electromagnetic effects, fumes from lubricants and heat accumulation from the moving parts of the equipment are a potential cause for concern. There are significant differences regarding light exposure, potential harm from moving parts and electromagnetic effects between the different systems. In the Primo Vision system, embryos are exposed to less light compared to the Embryoscope, and because there are no moving parts in the Primo Vision system, one does not have to worry about potential negative effects related to this issue. The light exposure of all systems avoids the use of short wavelength that is potentially detrimental to embryo development. In addition, the overall light exposure (in all available systems) is much lower than with standard observation when embryos have to be removed from the incubators and are exposed to light in the laboratory [33].

Several groups compared fertilization, embryo development, blastocyst formation and the implantation potential of embryos cultured in time-lapse versus standard incubation systems. Kirkegaard and colleagues [47] randomly assigned fertilized oocytes (676 oocytes from 59 patients) to time-lapse incubation/monitoring versus standard monitoring. Embryos were transferred on day 5. Selection for transfer was based on blastocyst morphology. Embryos in both groups were morphologically analyzed on days 2, 3 and 5 by removing them from the culture conditions. There was no difference in the cleavage rate and blastocyst formation rate between the two groups. Implantation and pregnancy rates did not differ either based on 19 and 18 transfers, respectively, in the two groups. The authors concluded that time-lapse incubation/ monitoring had no detrimental effects on embryo development.

To date, no negative impact regarding any of these parameters has been reported [17, 29, 36, 48]. The birth of the first healthy full term offspring following time-lapse incubation and selection based on time-lapse parameters was reported in 2010 [49], but more obstetric, neonatal and postnatal data are needed to establish to safety of the technology.

### Limitations of time lapse technology

The concept of continuous embryo observation improving IVF outcome seems sound at first look. The technology has been shown to exert no harmful effects on the embryos. The concept, however, has to be proven scientifically before routine clinical application can be recommended. The primary question is: are more observations better than a single daily observation for embryo selection?

This review has detailed several papers that have assessed the clinical utility of time-lapse monitoring. The reviewed studies already show promising results but suffer from methodological issues. First, essentially all of the cited studies have a retrospective design. Retrospective study design cannot account for differences in the patient populations or culturing conditions. It is well-known that patients with similar characteristics could have a different treatment outcome in different clinics. This fact has been shown in the study by Meseguer et al. [35] in which the impact of time-lapse monitoring on clinical pregnancy rate ranged between a few percent decrease to a 50% increase among the clinics participating in a multi-center trial. It is not known how much of this difference can be attributed to patient characteristics and how much to the different culture conditions. Culture conditions in a given lab (e.g., oxygen tension, culture medium used) could affect embryo development [50, 51]. The genetic integrity of the embryo is, however, expected to have an even more profound effect on the early development of the embryo. During embryo culture, the most crucial task is to differentiate embryos that will implant from those that will not. We can rephrase this statement and say that we need to differentiate the healthy, euploid embryos from the unhealthy, aneuploid embryos. Time-lapse technology has already shown us that euploid embryos follow a much tighter division pattern and aneuploid embryos tend to fall out of range [42]. Therefore, each lab should test whether the proposed kinetic parameters are appropriate for their lab or whether they need to modify them based on their own results rather than adopting them automatically.

It is also well-known that the treatment outcome depends on the stage at which embryo transfer occurs [28]. The different studies used different stage (day 2 to blastocyst stage) transfers [31, 36, 37], which may interfere with their conclusions regarding the kinetic markers due to their impact on implantation and pregnancy rates.

The currently proposed time-lapse parameters are not ready for routine clinical application yet because most of the published studies have not used clinically meaningful outcome parameters. Blastocyst formation and implantation rate are important surrogate markers of treatment efficacy, but neither can be used to replace the live birth rate or at least the ongoing pregnancy rate. Furthermore, in some of the discussed studies that are considered landmark studies [32, 42] in the field of time-lapse technology, embryos have not been transferred and therefore clinical data are not available. Another problem with the cited studies is that most of them draw conclusions based on small number of patients involved, as noted in a commentary by Ottolini et al. [46]. Data on the associations between ongoing pregnancy rate and live birth rate are limited at this stage. The above discussed studies involve mostly retrospective data analysis, and the time ranges for certain kinetic markers as well as the hierarchical models have not been properly tested prospectively.

Up until now, two RCTs have been published that compared standard incubation and embryo selection based on morphology with time-lapse incubation and morphokinetic embryo selection. Both trials included good prognosis patients or egg donation cycles. The larger study by Rubio et al. [41], reported an increase in the ongoing pregnancy rate among those couples who had their embryos cultured in the time-lapse system. In addition to the method of embryo selection (morphology alone vs morphokinetic markers based on various time-lapse parameters) there were significant differences in the culture conditions as well and this could have affected the results too. The proportion of good quality day 3 and day 5 embryos was significantly higher in the time-lapse system and this suggests more optimal incubation conditions. Due to differences in the culture conditions the exact role of morphokinetic selection in improving outcome cannot be determined. Both studies included good-prognosis patients only. Therefore, we can apply these results primarily in good prognosis patients. If the morphokinetic parameters are predictive of embryo health (and therefore implantation potential) then we should expect the models to work in a different subset of patients as well. In older patients or poor responders however, the proportion of embryos that are identified as having a higher implantation potential is expected to be lower though.

There are further ongoing trials that prospectively evaluate the benefits of time-lapse technology and their results should become available before a wider introduction into clinical care can be made [52].

## Conclusions

Time-lapse embryo observation allows us to monitor the dynamic events of embryo development as they happen rather than just evaluate snapshots of it. A lot has already been learned of the events of early embryonic development, and it has also been shown that if observations are made only once a day, some of the important changes the embryo undergoes (e.g., changes in fragmentation pattern) will be missed, which may result in the false identification of the best embryo for transfer [42, 53, 54].

While its full impact on clinical care needs to be explored, the technology could be useful for research and industry purposes as the steps of embryo development can be precisely standardized. Furthermore time-lapse technology could revolutionize quality control in the lab.

There is also a long way to go before the method’s routine application for embryo selection can be recommended. Certain parameters have already been identified that are associated with very low implantation potential. There are other markers that can predict blastocyst formation and implantation potential, though different groups have identified different markers. There is little data about the predictive ability of these parameters for clinical pregnancy and live birth (Table 3). A few hierarchical models (again based on different markers) have been proposed and tested in retrospective analyses [21, 32, 33]. The predictive ability of these markers has to be tested prospectively and using clinically meaningful endpoints.

**Table 3:** **The optimal time interval of kinetic markers predictive of different clinical outcomes by various groups**

	Wong et al. predictive of BC formation [32]	Meseguer et al. predictive of implantation [33]	Cruz et al. predictive of good morphology blastocyst development [35]	Conaghan et al. predictive of blastocyst formation [21]	Basile et al. predictive of euploidy [42]	Chavez et al. predictive of euploidy [41]	Campbell et al. predictive of euploidy [43], [44]
*S*_*1*_	14.3 ± 6 min					14.4 ± 4.2 min	
*CC*_*2*_	11.1 ± 2.2 h	≤11.9 h		9.33–11.45 h		11.8 ± 0.71 h	
*S*_*2*_	1 ± 1.6 h	≤0.76 h	≤ 0.76 h	≤1.73 h		0.96 ± 0.84 h	
*t*_*5*_		48.8–56.6 h	48.8–56.6 h		47.2–58.2 h		
*t*_*5-2*_					>20.5 h		
*CC*_*3*_					11.7–18.2 h		
*t*_*BC*_							<122.9 h (and <96.2 h time to start of blastulation)

S_1_: duration of first cytokinesis; CC_2_: t_3-2_;S_2_: t_4-3_;t_5_: time to 5-cell stage; CC_3_: t5-3; t_BC_: time to blastocyst development.

Thus far, the time-lapse technology has proven to be safe. However, pregnancy and neonatal outcome data must be collected as well.

Time-lapse technology is just one of the methods that is currently being evaluated for embryo selection. None of these technologies are perfect, and rather than looking at them as competing technologies, we should evaluate how they could complete each other and further improve embryo selection during IVF.

In summary, time-lapse technology provides us with a safe, undisturbed, continuous embryo observation option that can aid embryo selection and could be used for research purposes. However, the full benefit of the technology and its place among the other embryo screening tools remains to be determined.

## References

[1] Steptoe PCV, Edwards RG (1978). Birth after the reimplantation of human embryo. Lancet.

[2] Zegers-Hochschild F, Mansour R, Ishara O, Adamson GD, de Mouzon J, Nygren KG, Sullivan EA (2014). International Committee for Monitoring Assisted Reproductive Technology: world report on assisted reproductive technology, 2005. Fertil Steril.

[3] Ferraretti AP, Goossens V, Kupka M, Bhattacharya S, de Mouzon J, Castilla JA, Korsak V, Kupka M, Nygren KG, Nyboe Andersen A, European IVF-monitoring (EIM); Consortium for European Society of Human Reproduction and Embryology (ESHRE) (2013). Assisted reproductive technology in Europe, 2009: results generated from European registers by ESHRE. Hum Reprod.

[4] https://www.sartcorsonline.com/rptCSR_PublicMultYear.aspx?ClinicPKID=0

[5] Kovacs P (2012). Multiple pregnancies after ART and how to minimize their occurrence. Current Women’s Health Reviews.

[6] Practice Committee of the Society for Assisted Reproductive Technology and Practice Committee of the American Society for Reproductive Medicine (2012). Elective single-embryo transfer. Fertil Steril.

[7] Practice Committee of American Society for Reproductive Medicine; Practice Committee of Society for Assisted Reproductive Technology (2013). Criteria for number of embryos to transfer: a committee opinion. Fertil Steril.

[8] McLernon DJ, Harrild K, Bergh C, Davies MJ, de Neubourg D, Dumoulin JC, Gerris J, Kremer JA, Martikainen H, Mol BW, Norman RJ, Thurin-Kjellberg A, Tiitinen A, van Montfoort AP, van Peperstraten AM, Van Royen E, Bhattacharya S (2010). Clinical effectiveness of elective single versus double embryo transfer: meta-analysis of individual patient data from randomized trials. BMJ.

[9] Pandian Z, Marjoribanks J, Ozturk O, Serour G, Bhattacharya S (2013). Number of embryos for transfer following in vitro fertilisation or intra-cytoplasmic sperm injection. Cochrane Database Syst Rev.

[10] Staessen C, Platteau P, Van Assche E, Michiels A, Tournaye H, Camus M, Devroey P, Liebaers I, Van Steirteghem A (2004). Comparison of blastocyst transfer with or without preimplantation genetic diagnosis for aneuploidy screening in couples with advanced maternal age: a prospective randomized controlled trial. Hum Reprod.

[11] Mastenbroek S, Twisk M, van Echten-Arends J, Sikkema-Raddatz B, Korevaar JC, Verhoeve HR, Vogel NE, Arts EG, de Vries JW, Bossuyt PM, Buys CH, Heineman MJ, Repping S, van der Veen F (2007). In vitro fertilization with preimplantation genetic screening. N Engl J Med.

[12] Vergouw CG, Heymans MW, Hardarson T, Sfontouris IA, Economou KA, Ahlström A, Rogberg L, Lainas TG, Sakkas D, Kieslinger DC, Kostelijk EH, Hompes PG, Schats R, Lambalk CB (2014). No evidence that embryo selection by near-infrared spectroscopy in addition to morphology is able to improve live birth rates: results from an individual patient data meta-analysis. Hum Reprod.

[13] Vergouw CG, Kieslinger DC, Kostelijk EH, Botros LL, Schats R, Hompes PG, Sakkas D, Lambalk CB (2012). Day 3 embryo selection by metabolomic profiling of culture medium with near-infrared spectroscopy as an adjunct to morphology: a randomized controlled trial. Hum Reprod.

[14] Yang Z, Liu J, Collins GS, Salem SA, Liu X, Lyle SS, Peck AC, Sills ES, Salem RD (2012). Selection of single blastocysts for fresh transfer via standard morphology assessment alone and with array CGH for good prognosis IVF patients: results from a randomized pilot study. Mol Cytogenet.

[15] Scott RT, Upham KM, Forman EJ, Hong KH, Scott KL, Taylor D, Tao X, Treff NR (2013). Blastocyst biopsy with comprehensive chromosome screening and fresh embryo transfer significantly increases in vitro fertilization implantation and delivery rates: a randomized controlled trial. Fertil Steril.

[16] Payne D, Flaherty SP, Barry MF, Matthews CD (1997). Preliminary observations on polar body extrusion and pronuclear formation in human oocytes using time-lapse video cinematography. Hum Reprod.

[17] Mio Y, Maeda K (2008). Time-lapse cinematography of dynamic changes occurring during in vitro development of human embryos. Am J Obstet Gynecol.

[18] http://www.vitrolife.com/en/Fertility/Products/Primo-Vision-Time-Lapse-System/

[19] http://www.fertilitech.com/en-GB/Home.aspx

[20] http://www.eevaivf.com/

[21] Conaghan J, Cjhen AA, Willman SP, Ivani K, Chenette PE, Boostanfar R, Baker VL, Adamson GD, Abusief ME, Gvakharia M, Loewke KE, Shen S (2013). Improving embryo selection using a computer-automated time-lapse image analysis test plus day 3 morphology: results from a prospective multicenter trial. Fertil Steril.

[22] Alpha scientists in reproductive medicine and ESHRE special interest group of embryology (2011). The Istanbul consensus workshop on embryo assessments: proceedings of an expert meeting. Hum Reprod.

[23] Baczkowski T, Kurzawa R, Głabowski W (2004). Methods of embryo scoring in in vitro fertilization. Reprod Biol.

[24] van Montfoort AP, Dumoulin JC, Kester AD, Evers JL (2004). Early cleavage is a valuable addition to existing embryo selection parameters: a study using single embryo transfers. Hum Reprod.

[25] Borini A, Lagalla C, Cattoli M, Sereni E, Sciajno R, Flamigni C, Coticchio G (2005). Predictive factors for embryo implantation potential. Reprod Biomed Online.

[26] Källén B, Finnström O, Lindam A, Nilsson E, Nygren KG, Olausson PO (2010). Blastocyst versus cleavage stage transfer in in vitro fertilization: differences in neonatal outcome?. Fertil Steril.

[27] el Hajj N, Haaf T (2013). Epigenetic disturbances in in vitro cultured gametes and embryos: implications for human assisted reproduction. Fertil Steril.

[28] Glujovsky D, Blake D, Farquhar C, Bardach A (2012). Cleavage stage versus blastocyst stage embryo transfer in assisted reproductive technology. Cochrane Database Syst Rev.

[29] Kirkegaard K, Agerholm IE, Ingerslev HJ (2012). Time-lapse monitoring as a tool for clinical embryo assessment. Hum Reprod.

[30] Lemmen JG, Agerholm I, Ziebe S (2008). Kinetic markers of human embryo quality using time-lapse recordings of IVF/ICSI-fertilized oocytes. Reprod Biomed Online.

[31] Azzarello A, Hoest T, Mikkelsen AL (2012). The impact of pronuclei morphology and dynamicity on live birth outcome after time-lapse culture. Hum Reprod.

[32] Wong CC, Loewke KE, Bossert NI, Behr B, De Jonge CJ, Baer TM, Reijo Pera RA (2010). Non-invasive imaging of human embryos before embryonic genome activation predicts development to the blastocyst stage. Nat Biotechnol.

[33] Meseguer M, Herrero J, Tejera A, Hilligsoe KM, Ramsing N, Remohi J (2011). The use of morphokinetics as a predictor of embryo implantation. Hum Reprod.

[34] Rubio I, Kuhlmann R, Agerholm I, Kirk J, Herrero J, Escriba M-J, Bellver J, Meseguer M (2012). Limited implantation success of direct-cleaved human zygotes: a time-lapse study. Fertil Steril.

[35] Cruz M, Garrido N, Herrero J, Perez-Cano I, Munoz M, Meseguer M (2012). Timing of cell division in human cleavage-stage embryos is linked with blastocyst formation and quality. Reprod Biomed Online.

[36] Meseguer M, Rubio I, Cruz M, Basile N, Marcos J, Requena A (2012). Embryo incubation and selection in a time-lapse system improves pregnancy outcome compared with standard incubator: a retrospective cohort study. Fertil Steril.

[37] Chamayou S, Patrizio P, Storaci G, Tomaselli V, Alecci C, Ragolia C, Crescenzo C, Guglielmino A (2013). The use of morphokinetic parameters to select all emrbyos with full capacity to implant. J Assist Reprod Genet.

[38] Dal Canto M, Coticchio G, Mignini Renzini M, De Ponti E, Novara PV, Brambillasca F, Comi R, Fadini R (2012). Cleavage kinetics analysis of human embryos predicts development to blastocyst and implantation. Reprod Biomed Online.

[39] Kirkegaard K, Kesmodel US, Hindkjaer JJ, Ingerslev HJ (2013). Time-lapse parameters as predictors of blastocyst development and pregnancy outcome in embryos from good prognosis patients: a prospective cohort study. Hum Reprod.

[40] Kahraman S, Cetinkaya M, Pirkevi C, Yelke H, Kumtepe Y (2012). Comparison of blastocyst development and cycle outcome in patients with eSET using either conventional or time lapse incubators. A prospective study of good prognosis patients. J Reprod Stem Cll Biotechnol.

[41] Rubio I, Galán A, Larreategui Z, Ayerdi F, Bellver J, Herrero J, Meseguer M (2014). Clinical validation of embryo culture and selection by morphokinetic analysis: a randomized, controlled trial of the EmbryoScope. Fertil Steril.

[42] Chavez SL, Loewke KE, Han J, Moussavi F, Colls P, Munne S, Behr B, Reijo Pera RA (2012). Dynamic blastomere behavior reflect human embryo ploidy by the four-cell stage. Nat Commun.

[43] Basile N, del Carmen NM, Bronet F, Florensa M, Riquerios M, Rodrigo L, García-Velasco J, Meseguer M (2014). Increasing the probability of selecting chromosomally normal embryos by time-lapse morphokinetics analysis. Fertil Steril.

[44] Campbell A, Fishel S, Bowman N, Duffy S, Sedler M, Fontes Lindeman Hickman C (2013). Modelling a risk classification of aneuploidy in human embryos using non-invasive morphokinetics. Reprod Biomed Online.

[45] Campbell A, Fishel S, Bowman N, Duffy S, Sedler M, Thornton S (2013). Retrospective analysis of outcomes after IVF using an aneuploidy risk model derived from time-lapse imaging without PGS. Reprod Biomed Online.

[46] Ottolini C, Rienzi L, Capalbo A (2014). A cautionary note against embryo aneuploidy risk assessment using time-lapse imaging. Reprod Biomed Online.

[47] Kirkegaard K, Hindkjaer JJ, Grondahl ML, Kesmodel US, Ingerslev HJ (2012). A randomized clinical trial comparing embryo culture in a conventional incubator with a time-lapse incubator. J Assist Reprod Genet.

[48] Wong C, Chen AA, Behr B, Shen S (2013). Time-lapse microscopy and image analysis in basic and clinical embryo development research. Reprod Biomed Online.

[49] Pribenszky C, Mátyás S, Kovács P, Losonczi E, Zádori J, Vajta G (2010). Case report: pregnancy achieved by transfer of a single blastocyst selected by timelapse monitoring. Reprod Biomed Online.

[50] Kasterstein E, Strassburger D, Komarovsky D, Bern O, Komsky A, Raziel A, Friedler S, Ron-El R (2013). The effect of two distinct levels of oxygen concentration on embryo development in a sibling oocyte study. J Assist Reprod Genet.

[51] Ciray HN, Aksoy T, Goktas C, Ozturk B, Bahceci M (2012). Time-lapse evaluation of human embryo development in single versus sequential culture media – a sibling oocyt study. J Assist Reprod Genet.

[52] Kaser DJ, Racowsky C (2014). Clinical outcomes following selection of human preimplantation embryos with time-lapse monitoring: a systematic review. Hum Reprod Update.

[53] Pribenszky C, Losonczi E, Molnár M, Lang Z, Mátyás S, Rajczy K, Molnár K, Kovács P, Nagy P, Conceicao J, Vajta G (2010). Prediction of in-vitro developmental competence of early cleavage-stage mouse embryos with compact time-lapse equipment. Reprod Biomed Online.

[54] Hardarson T, Lofman C, Coull G, Sjogren G, Hamberger L, Edwards RG (2002). Internalization of cellular fragments in a human embryo: time-lapse recordings. Reprod Biomed Online.

